# Supplementary material for: Injectable Polypeptide Hydrogel Depots Containing Dual Immune Checkpoint Inhibitors and Doxorubicin for Improved Tumor Immunotherapy and Post-Surgical Tumor Treatment
Source: Pharmaceutics. 2023 Jan 28;15(2):428. doi: 10.3390/pharmaceutics15020428 (PMC9965187; doi:10.3390/pharmaceutics15020428)
Supplement: Supplementary file 1 [file pharmaceutics-15-00428-s001.zip › SR_pharmaceutics-2152766_Supplement.pdf]

Supplementary Materials: Injectable Polypeptide Hydrogel Depots Containing Dual Immune Checkpoint Inhibitors and Doxorubicin for Improved Tumor Immunotherapy and Post-Surgical Tumor Treatment

Zhixiong Chen, Yan Rong, Junfeng Ding, Xueliang Cheng, Xuesi Chen and Chaoliang He

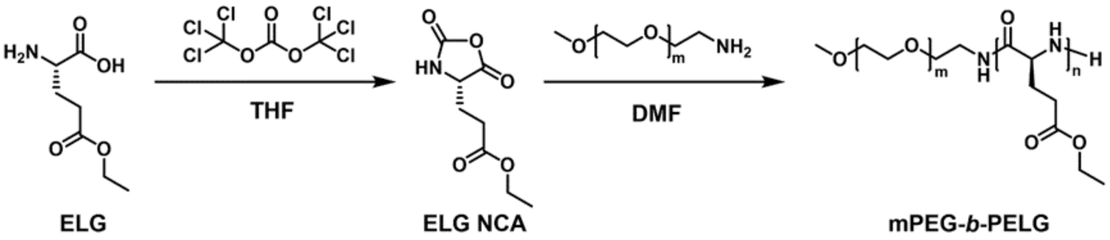

Scheme S1. Synthesis route for mPEG-PELG.

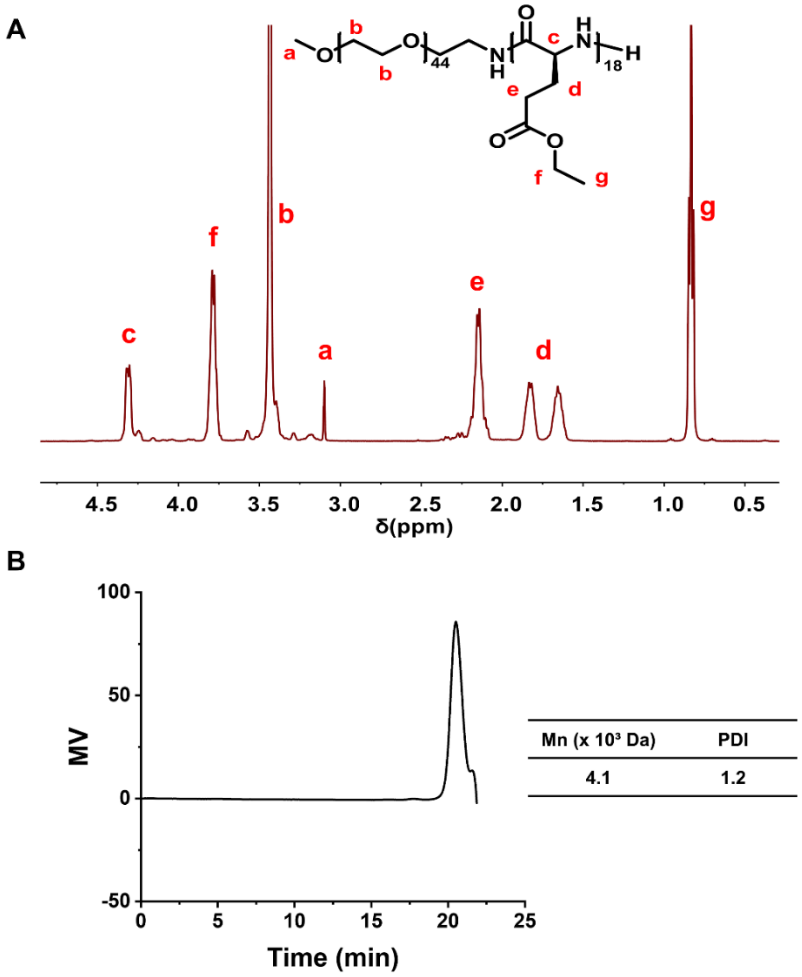

Figure S1. (A) 1H NMR spectrum of mPEG-PELG in CF3COOD. (B) GPC data of mPEG-PELG.

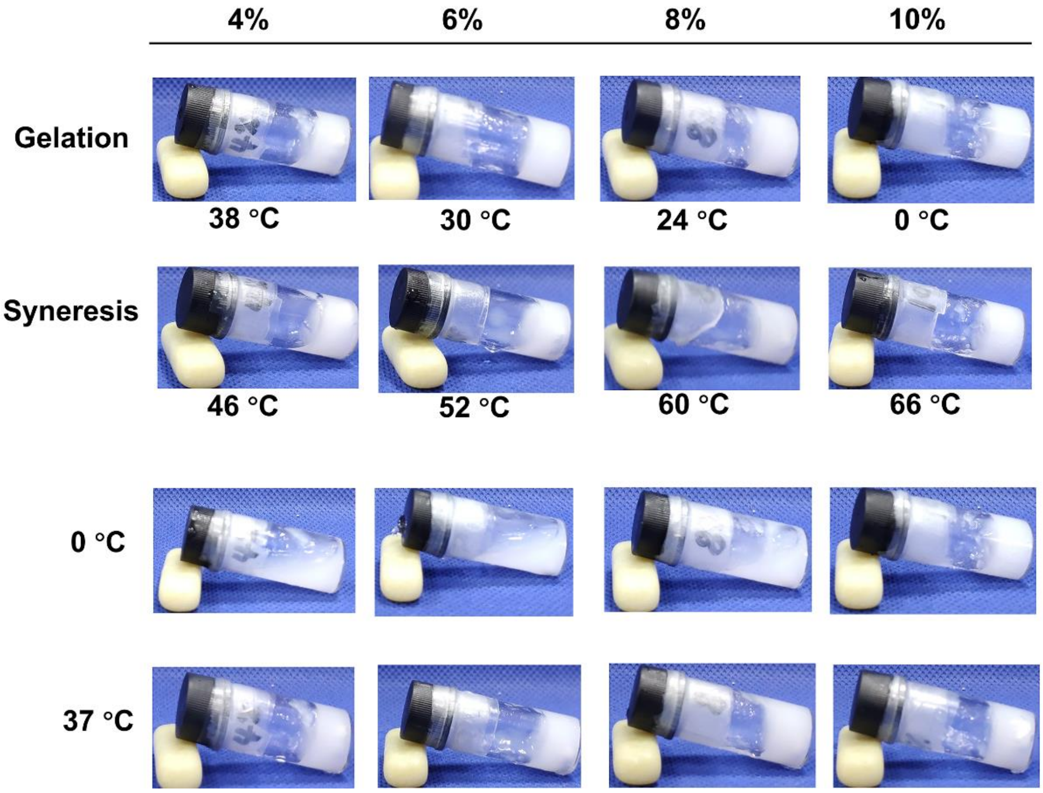

Figure S2. Photographs of the mPEG-PELG (4–10 wt%) at different temperatures. The gelation temperatures and syneresis temperatures were marked specially.

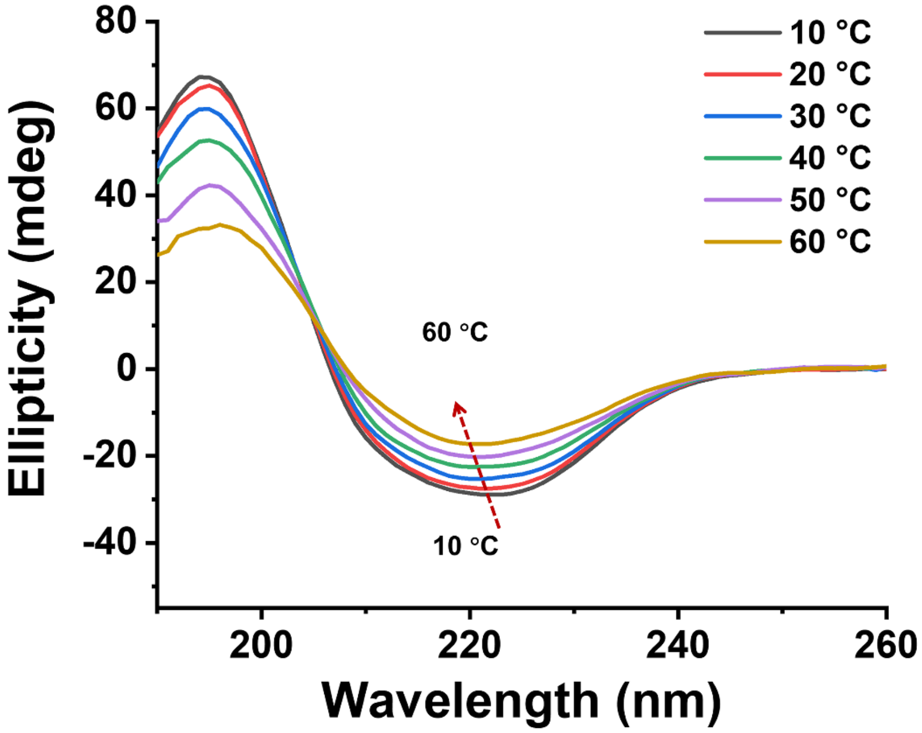

Figure S3. The ellipticity of polymer aqueous solution (0.05 wt%) with increasing temperature from 10 to 60 °C.

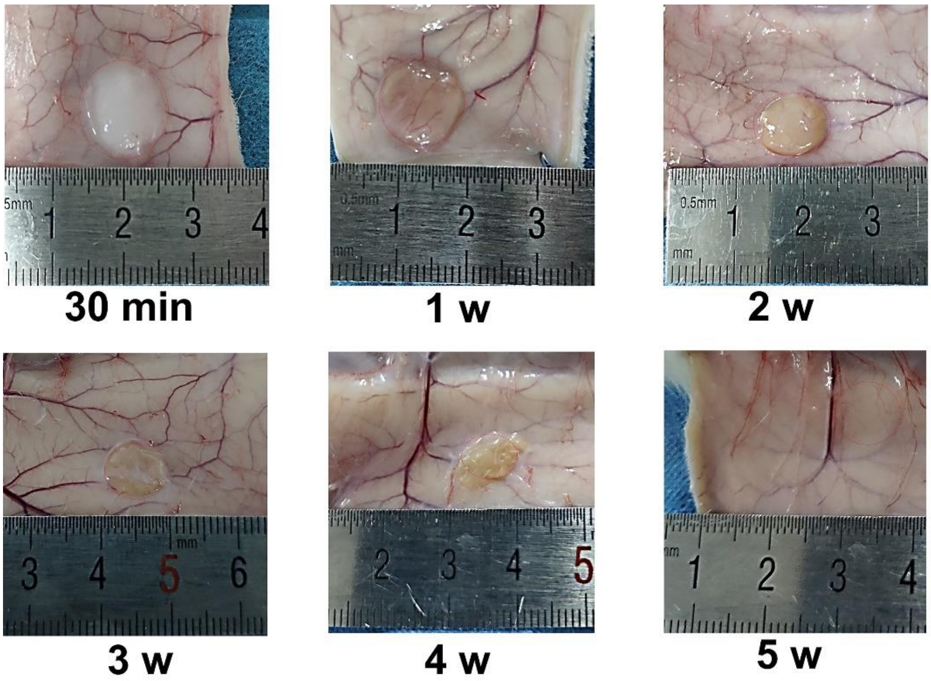

Figure S4. Photographs of the hydrogels after injection into the subcutaneous layer of rats at different time points.

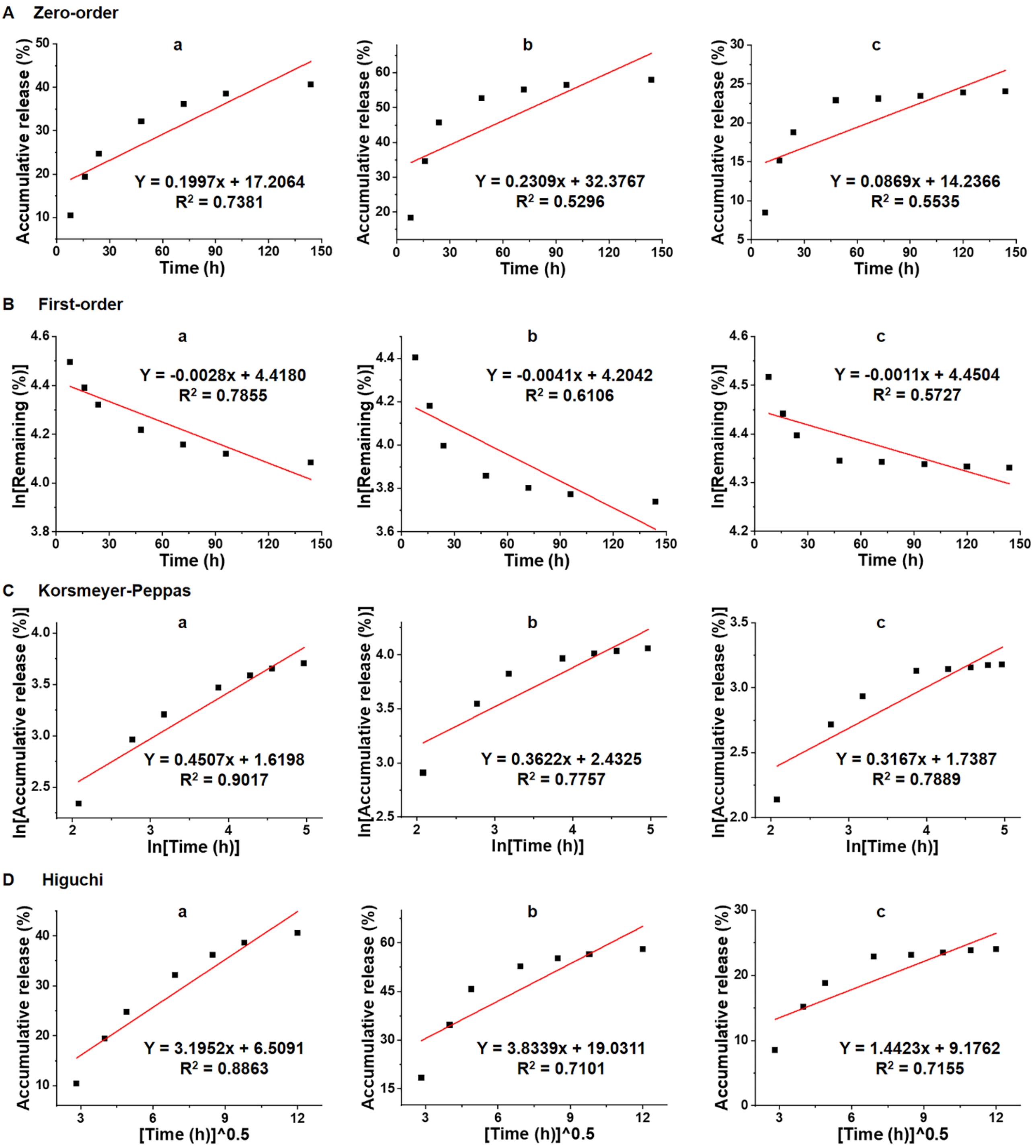

Figure S5. Fitting of the in vitro drug release data to kinetic models of zero-order model (A), first-order model (B), Korsmeyer-Peppas model (C) and Higuchi model (D) [43–45]. (a) Dox release from Dox-loaded hydrogel in PBS; (b) Dox release from Dox-loaded hydrogel in PBS containing proteinase K; (c) IgG release from IgG-loaded hydrogel in PBS.

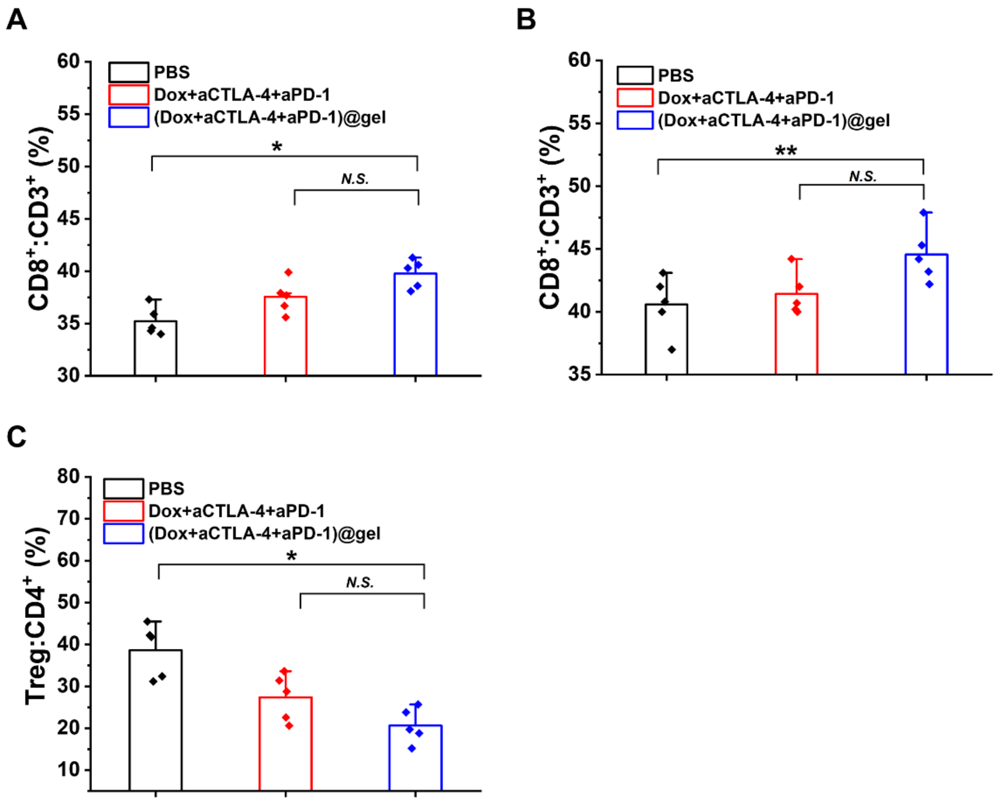

Figure S6. Immune cell analysis data of spleens, lymph nodes and tumors of the post-operative mice model after receiving various treatments, obtained by flow cytometry. (A) The ratio of $CD8^{+}$:$CD3^{+}$ in spleen (n = 5). (B) The ratio of $CD8^{+}$:$CD3^{+}$ in lymph nodes (n = 5). (C) The ratio of Tregs:$CD4^{+}$ in tumor (n = 5).
